# Identification of Strength-Development Pathways in One-Part Geopolymers Using ESI–MSI–LGP Indices and GMM Clustering

**DOI:** 10.3390/ma19143108

**Published:** 2026-07-20

**Authors:** Yiming Li, Zhenzhu Meng, Jian Huang, Yanwu Xiao, Zhibin Liu, Duo Zhang, Pinxue Chen

**Affiliations:** 1 School of Smart Urban Construction, Guangzhou City Polytechnic, Guangzhou 511370, China; ymli@gcp.edu.cn (Y.L.); xyw@gcp.edu.cn (Y.X.); chenpinxue@gcp.edu.cn (P.C.); 2 School of Hydraulic Engineering, Zhejiang University of Water Resources and Electric Power, Hangzhou 310018, China; 3 School of Civil Engineering and Architecture, Anyang Normal University, Anyang 455000, China; zhibinliu02155@163.com; 4 School of Civil Engineering, Sun Yat-sen University, Zhuhai 519082, China

**Keywords:** OPG, one-part geopolymer, clustering, strength development, Gaussian mixture model

## Abstract

Geopolymer research has usually focused on predicting compressive strength at specific curing ages, while the underlying strength-development behavior remains insufficiently understood. To address this gap, this study proposes a pathway-oriented framework for characterizing strength-development patterns in one-part geopolymers (OPGs). A compiled experimental database was established from published studies, and three dimensionless indices, namely the Early Strength Index (ESI), Mid-age Strength Index (MSI), and Late-stage Growth Potential (LGP), were introduced to quantify temporal strength-development behavior. Then, Gaussian Mixture Model (GMM) clustering was employed in the ESI–MSI–LGP feature space to identify latent strength-development pathways. Based on the available core dataset, three representative strength-development pathways were identified: Fast-hardening, Delayed-hardening, and Balanced-hardening. The Fast-hardening pathway exhibited the highest average ESI and MSI values, indicating rapid early- and mid-age strength attainment. The Delayed-hardening pathway showed lower ESI and MSI values but the highest LGP value, reflecting stronger later-age strength-growth potential after 7 days. The Balanced-hardening pathway was characterized by relatively low ESI, high MSI, and low LGP, suggesting accelerated strength development between 3 and 7 days. Further analysis of mix-design variables indicated that pathway formation was associated with the coupled effects of aggregate-to-binder proportion, activator chemistry, water availability, precursor composition, and curing conditions, rather than being controlled by a single dominant factor. Finally, a smooth probability-based strength-development map was established in the ESI–MSI feature space, providing a practical visualization tool for approximate pathway identification and preliminary mixture-selection support. The proposed framework shifts the focus from conventional strength prediction toward strength-development behavior characterization and offers new insights into the macroscopic hardening patterns of one-part geopolymers.

## 1. Introduction

The cement industry is a major contributor to global anthropogenic CO_2_ emissions, which has motivated the development of alternative low-carbon cementitious materials [1,2,3]. Geopolymers and alkali-activated materials are promising candidates because they can utilize industrial by-products and waste materials while maintaining satisfactory mechanical and durability performance [4,5]. Among them, one-part geopolymers (OPGs), also known as ‘just-add-water’ geopolymers, are particularly attractive because they avoid the handling of highly alkaline liquid activators [6,7,8].

Over the past decade, extensive studies have investigated the mechanical performance, reaction mechanisms, and microstructural evolution of OPG systems. Early research confirmed the feasibility of producing fly ash- and slag-based one-part geopolymer binders with satisfactory compressive strength under different curing conditions [6,7]. Subsequent studies further showed that precursor composition, slag incorporation, activator chemistry, water availability, and curing conditions strongly affect the fresh properties, strength development, and reaction kinetics of OPGs [8,9,10,11,12,13,14]. More recently, various industrial by-products and solid wastes, including coal gasification slag, biomass ash, phosphogypsum, molybdenum tailings, and bauxite residue, have been explored as alternative precursors for sustainable OPG systems [15,16,17,18,19]. These findings collectively indicate that the compressive strength and hardening behavior of OPGs are governed by coupled effects among precursor characteristics, activator composition, water content, and curing conditions [20,21,22,23].

In parallel with experimental investigations, considerable efforts have been devoted to mixture optimization and performance enhancement. Statistical and response-surface methodologies have been employed to identify optimal mixture proportions and evaluate the relationships among design variables [24,25]. For example, Hang et al. (2023) [26] analyzed the correlations between key design factors and compressive strength, whereas Faridmehr et al. (2023) [27] optimized fly ash–slag OPG systems to improve engineering performance. Similar optimization-oriented studies have also been reported for ambient-cured high-performance OPGs and geopolymer mortars [28,29]. These investigations have improved the understanding of how mixture-design parameters affect the performance of OPGs.

More recently, the rapid development of artificial intelligence has provided new opportunities for modeling and prediction [30,31,32,33]. Various machine learning algorithms, such as support vector machines, ensemble learning methods, random forests, artificial neural networks, gradient boosting algorithms, and deep learning frameworks, have been successfully applied to predict the compressive strength of geopolymer materials [34,35]. For instance, Wei et al. (2024) [35] combined experimental investigations with machine learning methods to analyze the compressive strength behavior of one-part geopolymers. Wang et al. (2025) further employed interpretable machine learning methods to identify the dominant variables governing compressive strength in OPG systems [36]. Similar data-driven frameworks have been extended to one-part geopolymer-stabilized soils for predicting unconfined compressive strength and freeze–thaw resistance [37,38,39]. Recent studies have also highlighted the importance of explainable machine learning in understanding material behavior beyond simple prediction accuracy [40,41].

Despite these advances, most existing studies focus primarily on predicting compressive strength at specific curing ages, particularly the 28-day strength. Strength values measured at different ages are typically treated as independent response variables, while the temporal evolution of strength is rarely investigated as an integrated hardening process [34,35,36]. Consequently, mixtures exhibiting similar 28-day strengths may follow substantially different strength-development trajectories. The existing literature therefore lacks a systematic framework for characterizing, classifying, and interpreting latent hardening pathways in OPG systems. Identifying such pathways could provide new insights into the mechanisms governing strength evolution and support more rational mixture-selection support.

To address these knowledge gaps, this study proposes a pathway-oriented data-driven framework for identifying and interpreting strength-development behaviors in one-part geopolymers. A comprehensive database was compiled from published studies, covering mixture-design variables, curing conditions, and multi-age compressive strength records. Three dimensionless indices, namely the Early Strength Index (ESI), Mid-age Strength Index (MSI), and Late-stage Growth Potential (LGP), were introduced to characterize different aspects of temporal strength evolution. As the strength-development behaviors of OPGs are potentially heterogeneous and not known a priori, unsupervised clustering provides a suitable means to discover latent pathway structures without imposing predefined labels [42,43,44]. Based on the core samples containing complete 3-day, 7-day, and 28-day compressive strength records, Gaussian Mixture Model (GMM) clustering was employed to identify latent strength-development pathways in the ESI–MSI–LGP feature space [45,46]. Interpretable machine learning and statistical analyses were further used to investigate the relationships between pathway labels and mixture-design variables, leading to the development of a practical strength-development map for pathway identification and mixture optimization. The main contributions of this study are summarized as follows:

(1) A pathway-oriented strength-development characterization framework is proposed for one-part geopolymers. Three dimensionless indices, namely ESI, MSI, and LGP, are introduced to quantify early-age strength attainment, mid-age strength attainment, and later-age growth potential. Based on these indices, GMM clustering is employed in the ESI–MSI–LGP feature space to identify three representative strength-development pathways: Fast-hardening, Delayed-hardening, and Balanced-hardening.

(2) The relationships between the representative pathways and mixture-design variables are examined through statistical comparison and interpretable machine learning analysis. A smooth probability-based strength-development map is further constructed in the ESI–MSI feature space, providing a practical visualization tool for approximate pathway identification and preliminary mixture-selection support.

## 2. Materials and Dataset

### 2.1. Raw Materials and Dataset Sources

**Figure 1:**
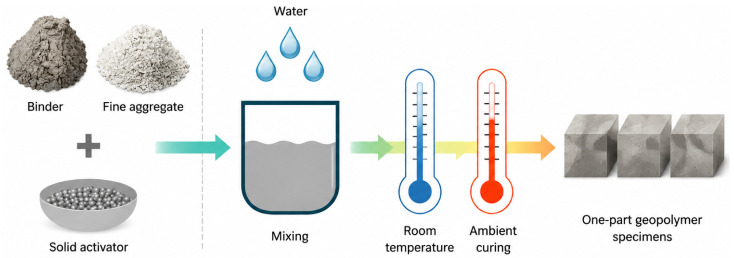
Flow of raw material preparation and curing processes for one-part geopolymers.

One-part geopolymers are typically prepared by dry-mixing aluminosilicate precursors with solid alkaline activators, followed by the addition of water to initiate dissolution, geopolymerization, setting, and hardening. Compared with conventional two-part geopolymer systems that require pre-prepared liquid alkaline solutions, one-part geopolymers offer a more convenient “just-add-water” preparation route, which is closer to ordinary Portland cement-based materials in terms of handling and field application. Figure 1 illustrates the general preparation and curing process for one-part geopolymers.

To investigate the strength-development behavior of one-part geopolymers, a compiled database was established from previously published experimental studies. The database contains 183 experimental records collected from 26 literature sources [6,7,8,9,10,11,12,13,14,17,20,21,22,23,24,25,28,29,35,47,48,49,50,51,52,53]. These studies cover different precursor proportions, alkali contents, activator moduli, sand-to-binder ratios, curing conditions, etc., thereby providing a broad basis for analyzing the strength-development pathways of one-part geopolymers.

### 2.2. Database Description

The compiled database includes seven input variables and four compressive strength outputs. The input variables are slag content (Slag, %), Na_2_O dosage (Na_2_O, %), sand-to-binder ratio (s/b), water-to-binder ratio (w/b), activator modulus ($M_{s}$), curing temperature (Temperature, °C), and curing humidity (Humidity, -). Humidity was represented as normalized relative humidity on a 0–1 scale, with higher values denoting higher-humidity curing and lower values denoting ambient low-humidity curing. The output variables are compressive strengths at 3, 7, 14, and 28 days, denoted as CS_3d_, CS_7d_, CS_14d_, and CS_28d_, respectively.

**Figure 2:**
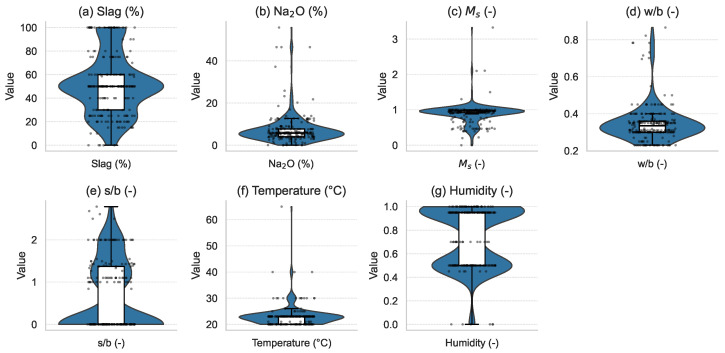
Statistical distributions of the seven input variables in the compiled one-part geopolymer database.

Figure 2 shows the statistical distributions of the seven input variables. The slag content covers the full range from 0% to 100%, with a mean value of 50.894%, indicating that the database includes low-slag, high-slag, and blended fly ash–slag systems. Na_2_O dosage ranges from 0% to 55.8%, with a mean value of 7.334%, while most samples are concentrated at relatively low alkali contents. The activator modulus $M_{s}$ ranges from 0 to 3.326, with an average value of 0.895, and most values are concentrated around 0.8–1.2. The w/b ratio ranges from 0.23 to 0.866, with a mean value of 0.349, reflecting the typical low-water demand of one-part geopolymer mixtures. The s/b ratio ranges from 0 to 2.786, indicating that the database includes both paste-like and mortar-like systems. The curing conditions are also summarized in the database. The curing temperature ranges from 20 to 65 °C, with a mean value of 22.986 °C, suggesting that most specimens were cured under ambient or near-ambient conditions, while a limited number of thermally cured cases were also included. The humidity ranges from 0 to 1, with a mean value of 0.740, covering both relatively dry and high-humidity curing environments.

The summary statistics of the compiled database are listed in Table 1. The 28-day compressive strength covers a wide range from 0 to 105 MPa, with a mean value of 48.903 MPa and a standard deviation of 25.433 MPa. Its large variation indicates that the database includes one-part geopolymer mixtures with substantially different mechanical performance levels. The average compressive strengths at 3 and 7 days are 30.833 MPa and 37.353 MPa, respectively. Compared with the mean 28-day strength, these values correspond to approximately 63% and 76% of the 28-day strength, indicating that one-part geopolymers generally exhibit pronounced early-age strength development. This feature provides a strong basis for further characterizing different hardening pathways.

## 3. Methods

**Figure 3:**
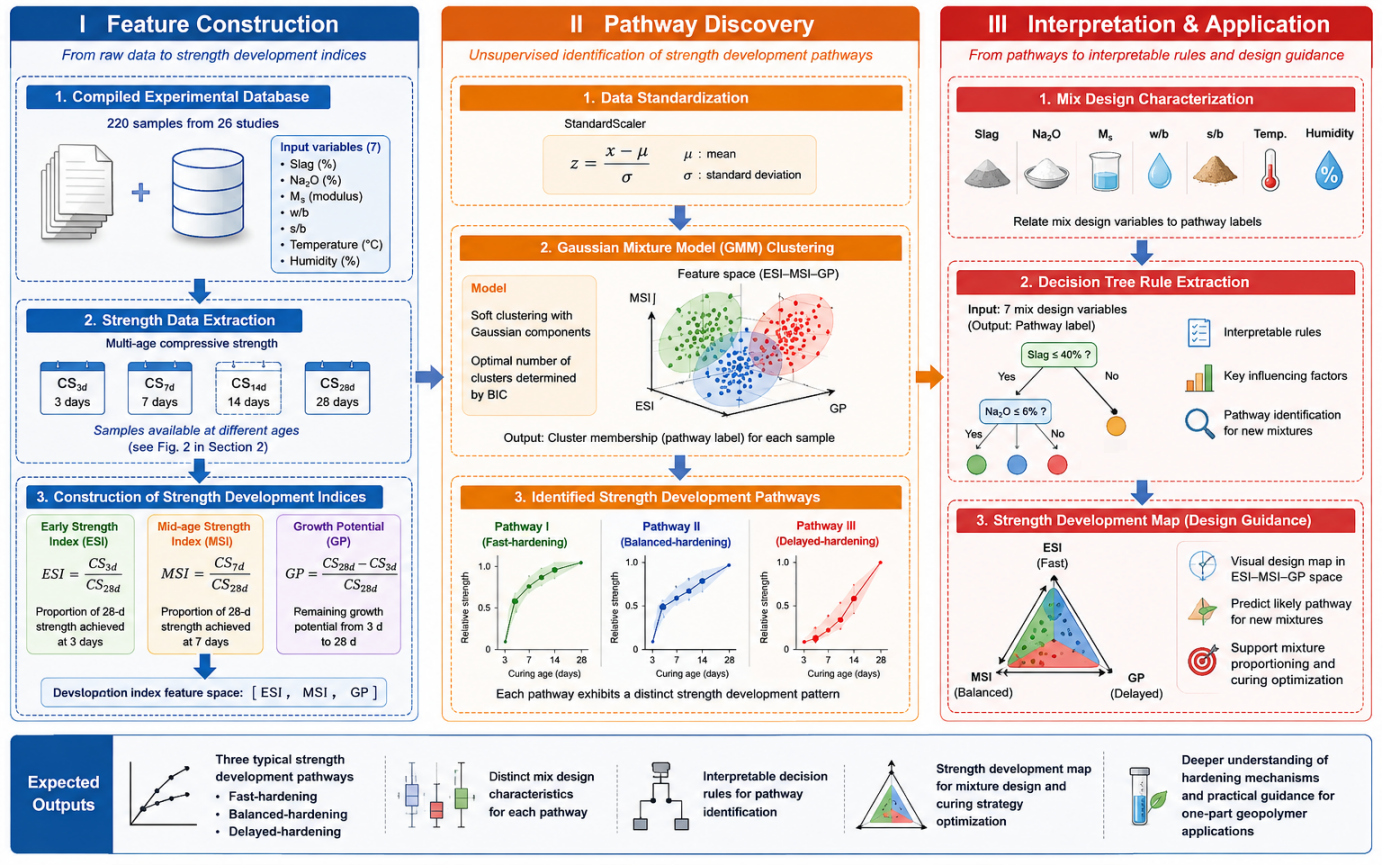
Overall methodological framework for identifying strength-development pathways in one-part geopolymers.

Figure 3 presents the overall methodological framework adopted in this study. The framework consists of three major stages, namely feature construction, pathway discovery, and interpretation and application. First, multi-age compressive strength data were transformed into dimensionless strength-development indices. Subsequently, Gaussian Mixture Model (GMM) clustering was employed to identify distinct strength-development pathways. Finally, the relationships between pathway labels and mix-design variables were interpreted using a decision tree (DT) model, leading to the development of a practical strength-development map for one-part geopolymers. ChatGPT 5.5 (OpenAI) was used to assist in visually rendering the author-developed textual framework for Figure 3.

### 3.1. Construction of Strength Development Indices

The strength development of one-part geopolymers is commonly evaluated using compressive strengths measured at different curing ages. However, the direct use of compressive strength values may be affected by the absolute strength level of individual mixtures. To characterize the relative hardening behavior independent of the final strength magnitude, three dimensionless strength-development indices were proposed.

The first indicator is the Early Strength Index (ESI), defined as

(1) $$ESI=\frac{CS_{3d}}{CS_{28d}}\;$$

where $CS_{3d}$ and $CS_{28d}$ denote the compressive strengths measured at 3 and 28 days, respectively.

The ESI quantifies the proportion of the 28-day strength achieved during the first three days of curing. Higher ESI values indicate rapid early-age hardening.

The second indicator is the Mid-age Strength Index (MSI), defined as

(2) $$MSI=\frac{CS_{7d}}{CS_{28d}}\;$$

where $CS_{7d}$ denotes the compressive strength at 7 days.

The MSI reflects the degree of strength development achieved within the first week and represents the intermediate-stage hardening efficiency.

The third indicator is the Late-stage Growth Potential (LGP), defined as

(3) $$LGP=\frac{CS_{28d}-CS_{7d}}{CS_{28d}-CS_{3d}}\;$$

The LGP quantifies the proportion of post-3-day strength gain that occurs between 7 and 28 days. A larger LGP value indicates that a larger share of the strength increment from 3 to 28 days occurs after 7 days, reflecting stronger LGP.

Prior to index calculation, zero-coded or missing strength measurements were treated as unavailable data. Records without complete and positive CS_3d_, CS_7d_, and CS_28d_ measurements were excluded from the core dataset. Records with CS_28d_ ≤ CS_3d_ or non-finite index values were also specified for exclusion to avoid undefined ratios and unstable LGP values.

Consequently, each mixture can be represented by the following three-dimensional strength-development feature vector:

(4) X=[ESI,MSI,LGP]

This feature space serves as the basis for subsequent pathway identification.

### 3.2. Identification of Strength Development Pathways

Prior to clustering, all strength-development indices were standardized using the z-score transformation to eliminate the influence of different numerical scales.

(5) $$z_{i}=\frac{x_{i}-\mu }{\sigma }\;$$

where $x_{i}$ is the original feature value, μ is the feature mean, and σ is the corresponding standard deviation.

The standardized feature vectors were then analyzed using the Gaussian Mixture Model (GMM). Unlike conventional partitioning methods such as K-means, the GMM provides a probabilistic clustering framework capable of describing overlapping data distributions frequently observed in material datasets.

The probability density function of the GMM can be expressed as

(6) $$p\left(x\right)=\sum_{k=1}^{K}\pi_{k}N\left(x|\mu_{k},\Sigma_{k}\right)\;$$

where *K* is the number of Gaussian components, $\pi_{k}$ is the mixing coefficient, and $N(x|\mu_{k},\Sigma_{k})$ denotes the Gaussian distribution characterized by mean vector $\mu_{k}$ and covariance matrix $\Sigma_{k}$.

The optimal number of clusters was evaluated using both the Akaike Information Criterion (AIC) and the Bayesian Information Criterion (BIC). The silhouette coefficient was used to evaluate cluster separation. Lower AIC and BIC values indicate better trade-offs between model fit and model complexity. A larger silhouette coefficient indicates better cluster separation. Considering the limited sample size, the final value of *K* was determined by jointly considering statistical performance, solution stability, and physical interpretability. The consistency between the resulting partitions and the adopted clustering solution was quantified using the adjusted Rand index (ARI).

**Figure 4:**
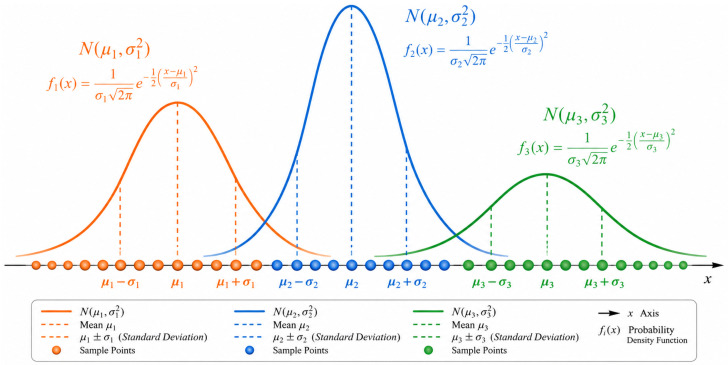
The schematic representation of the GMM.

Each cluster obtained from the GMM represents a distinct strength-development pathway characterized by unique combinations of ESI, MSI, and LGP values. To visualize the spatial distribution and overlap of the identified pathways, kernel density estimation (KDE) was applied in the ESI–MSI feature space. The schematic representation of the GMM clustering is illustrated in Figure 4.

Algorithm 1 summarizes the pseudocode of the proposed GMM-based procedure for identifying strength-development pathways.
**Algorithm 1** GMM-based identification of strength-development pathways**Require:** Multi-age compressive strength dataset D**Ensure:** Pathway labels y and optimal number of clusters $K^{*}$ 1:Compute ESI, MSI, and LGP according to Equations (1)–(3) 2:Remove samples with incomplete ESI, MSI, or LGP values 3:Obtain the core feature matrixX=[ESI,MSI,LGP] 4:Standardize X using z-score normalization to obtain Z 5:**for** K=2 to 10 **do** 6:      Fit a GMM with *K* Gaussian components to Z 7:      Compute AIC(K), BIC(K), and silhouette coefficient S(K) 8:**end for** 9:Select the optimal number of clusters $K^{*}$ by jointly considering AIC, BIC, S(K), and physical interpretability10:Fit the final GMM model with $K^{*}$ components11:Assign each sample to a pathway label:       $$\;y_{i}=\arg\underset{k}{\max}P\left(k|z_{i}\right)$$12:Use the pathway labels for statistical characterization, KDE visualization, and decision tree interpretation**return** y, $K^{*}$

### 3.3. Mix-Design Characterization and Rule Extraction

After pathway identification, the relationships between pathway labels and mix-design variables were further investigated. To establish interpretable pathway-classification rules, a decision tree (DT) model was employed. The pathway labels generated by the GMM clustering served as the target variable, while the seven mix-design variables were adopted as input features. Differences in the mixture-design variables among the three pathways were evaluated using the Kruskal–Wallis test, with p<0.05 considered statistically significant. One-way analysis of variance (ANOVA) was additionally performed as a parametric sensitivity comparison.

The DT recursively partitions the feature space by maximizing the reduction in node impurity. The Gini impurity criterion was adopted in this study:

(7) $$Gini=1-\sum_{i=1}^{C}p_{i}^{2}$$

where $p_{i}$ is the probability of class *i* and *C* denotes the total number of pathway categories. To reduce overfitting, the decision tree was restricted to a maximum depth of 2, with a minimum of five samples per leaf and a cost-complexity pruning parameter of 0.005. The dataset was divided using a stratified 70/30 training–testing split, and model performance was evaluated using accuracy, precision, recall, F1-score, and the confusion matrix. The resulting decision tree provides simplified threshold-based rules for exploring possible associations between mix-design variables and pathway labels. These rules are used for interpretation rather than deterministic pathway prediction.

### 3.4. Construction of the Strength-Development Map

To construct a two-dimensional visualization of the identified pathways, an auxiliary support vector classifier with a radial basis function kernel (RBF-SVC) was trained using ESI and MSI as input variables and the GMM-derived pathway labels as targets. The input variables were standardized before model training, and probability estimation was enabled to generate smooth pathway-probability regions. The trained classifier was then applied to a regular grid over the ESI–MSI domain, with each grid point assigned to the pathway having the highest estimated probability.

The original pathway labels were obtained from GMM clustering in the three-dimensional ESI–MSI–LGP feature space. Therefore, the resulting map should be interpreted as a two-dimensional projection-based visualization rather than a complete representation of the original GMM clustering boundaries. The auxiliary RBF-SVC was used only for visualization, whereas the decision tree described in the preceding section was used separately to explore associations between mixture-design variables and the GMM-derived pathway labels.

## 4. Results

### 4.1. Characteristics of Strength Development Indices

**Figure 5:**
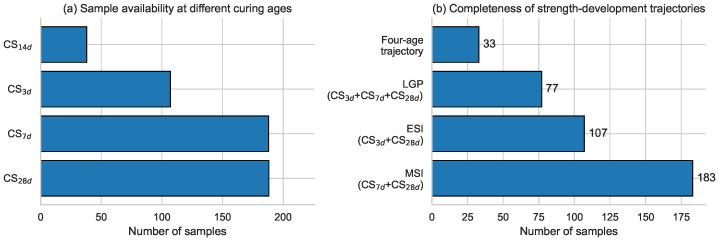
Availability and completeness of compressive strength data for strength-development analysis.

Since the objective of this study is to identify strength-development pathways rather than merely predict compressive strength at a single curing age, the completeness of multi-age strength data is critical. Figure 5 summarizes the data availability for constructing the proposed strength-development indices. Among the three indices, MSI can be calculated for 180 samples with paired 7-day and 28-day strength records, while ESI can be calculated for 107 samples with paired 3-day and 28-day strength records. The newly defined LGP requires simultaneous 3-day, 7-day, and 28-day strength measurements, and is therefore available for 77 samples. Consequently, these 77 samples constitute the core dataset used for the subsequent pathway identification, characterization, and interpretable modeling analyses. All 77 core records satisfied CS_3d_ ≤ CS_7d_ ≤ CS_28d_, and no negative or greater-than-unity LGP values were observed. Therefore, none of the complete records were removed because of non-monotonic strength development.

The 77-record core subset should not be interpreted as a statistically representative sample of the entire database. Compared with records lacking complete three-age measurements, the subset contains relatively greater representation of slag-rich, higher-alkali, and high-humidity mixtures, and fewer high-sand and elevated-temperature cases. This difference reflects the non-random availability of multi-age strength data in the literature and may influence the relative sizes and characteristics of the identified pathways.

**Figure 6:**
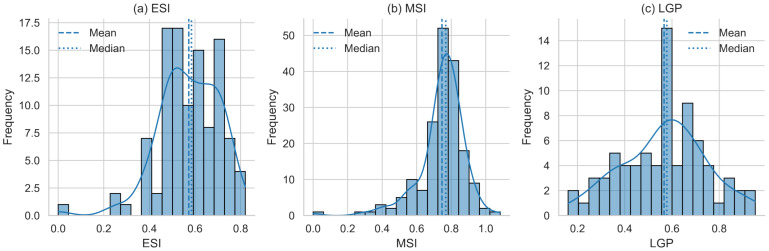
Statistical distributions of the strength-development indices.

To further examine the statistical characteristics of the proposed indices, their distributions are presented in Figure 6. The distributions of ESI were calculated using the 107 samples containing both 3-day and 28-day compressive strength measurements, whereas MSI was calculated using the 183 samples containing both 7-day and 28-day strength data. The LGP values were calculated from the 77 core samples containing complete 3-day, 7-day, and 28-day strength records.

The ESI values are primarily distributed between approximately 0.40 and 0.75, with both the mean and median located near 0.58, indicating that nearly 60% of the 28-day compressive strength is achieved within the first three days of curing on average. In comparison, MSI exhibits a narrower distribution concentrated between approximately 0.65 and 0.90, with mean and median values close to 0.76, suggesting that most mixtures attain about three-quarters of their 28-day strength within the first week. The LGP values are mainly distributed between approximately 0.35 and 0.75, indicating that the relative contribution of later-age strength gain after 7 days varies considerably among different mixtures.

Overall, the proposed indices exhibit broad yet well-defined distributions, reflecting substantial variability in strength-development behavior among one-part geopolymer mixtures. ESI and MSI describe early-age and mid-age strength attainment, respectively, whereas LGP further characterizes the proportion of post-3-day strength gain that occurs after 7 days. These differences suggest that the database may contain multiple latent hardening behaviors rather than a single homogeneous strength-development pattern, providing a basis for the subsequent clustering analysis.

### 4.2. Identification of Strength Development Pathways

**Figure 7:**
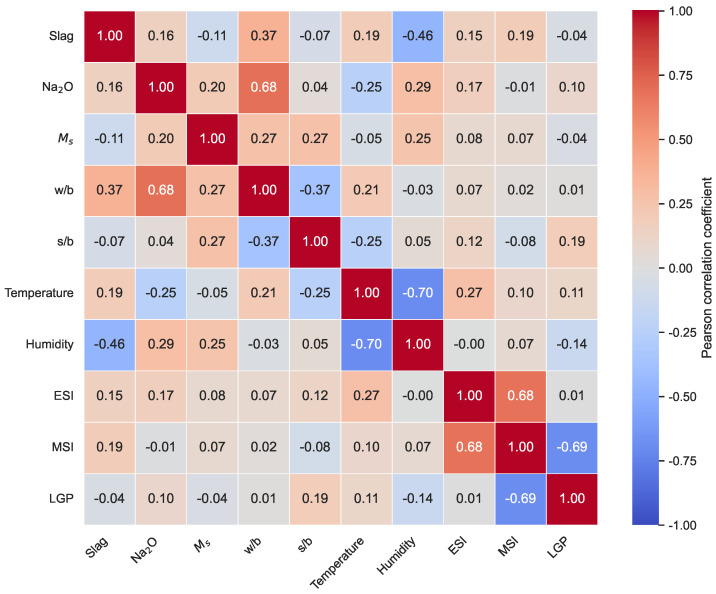
Pearson correlation matrix between mix-design variables and strength-development indices (ESI, MSI, and LGP) based on the 77 core samples used for pathway identification.

Before performing GMM clustering, the correlation structure of the 77 core samples was examined. Figure 7 presents the Pearson correlation matrix between the seven mix-design variables and the three strength-development indices, namely the Early Strength Index (ESI), Mid-age Strength Index (MSI), and Late-stage Growth Potential (LGP). Overall, the correlations between individual mix-design variables and the development indices were weak. Temperature showed the highest correlation with ESI (r=0.27), suggesting a possible effect of curing temperature on early-age hardening. Slag content was weakly correlated with ESI (r=0.15) and MSI (r=0.19), while s/b showed a weak correlation with LGP (r=0.19). The other mix-design variables exhibited limited linear correlations with the three indices.

Among the development indices, ESI and MSI showed a relatively strong positive correlation (r=0.68), indicating that mixtures with higher early-age strength ratios generally also exhibit higher 7-day strength ratios. In contrast, LGP was negatively correlated with MSI (r=−0.69), suggesting that mixtures with greater mid-age strength development tend to have lower later-age growth potential. Notably, LGP showed almost no correlation with ESI (r=0.01), indicating that the revised LGP provides additional information beyond the early-strength ratio. These results support the use of the ESI–MSI–LGP feature space for identifying latent strength-development pathways.

**Figure 8:**
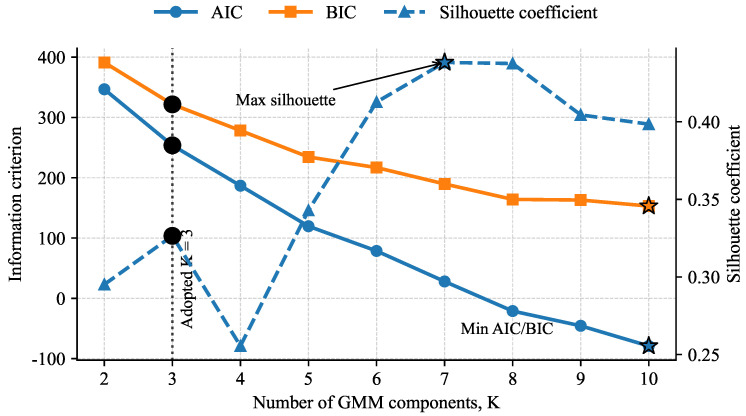
Variation in AIC, BIC and silhouette coefficient with the number of GMM clusters.

To identify distinct strength-development behaviors, GMM clustering was performed using the standardized ESI, MSI, and LGP features described in Section 3. After removing samples with incomplete strength-development records, 77 mixtures containing complete 3-day, 7-day, and 28-day compressive strength data were retained for clustering analysis. The AIC, BIC, and silhouette coefficient were used to evaluate the clustering results with different numbers of GMM components, as shown in Figure 8. The AIC and BIC values generally decreased as the number of components increased, while the highest silhouette coefficient was obtained at a larger cluster number. Although higher K values improved the statistical partitioning metrics, the K = 4 and K = 5 solutions mainly produced smaller subdivisions of the same broad hardening regions rather than clearly distinct development pathways. Given the core sample size, these finer solutions would result in sparsely populated subclasses with limited physical interpretability. In addition, the repeated-initialization analysis for K = 3 yielded a mean ARI of 0.887 and a median ARI of 1.000, with 14 of 20 runs reproducing the adopted partition. Therefore, K = 3 was selected as a coarse-grained representation that balances statistical fit, stability, sample size, and physical interpretability.

**Figure 9:**
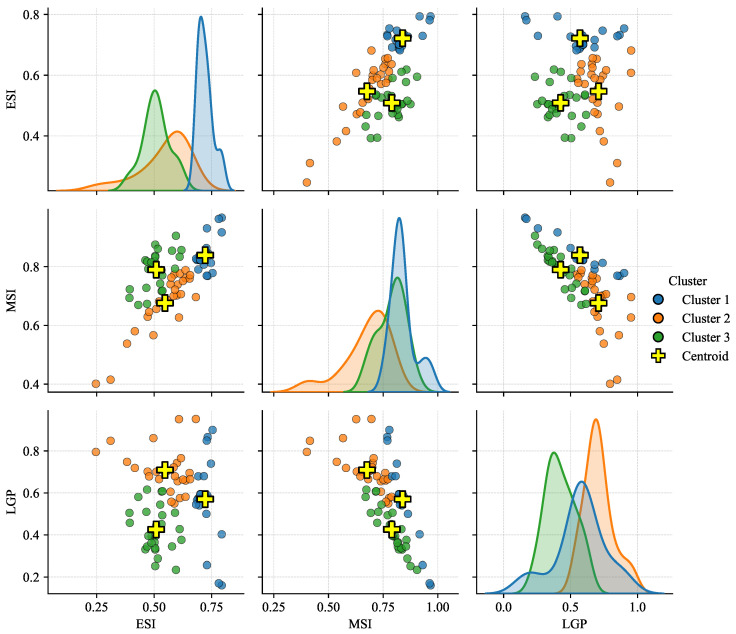
Pairwise projections of strength-development pathways identified by GMM clustering in the ESI–MSI–LGP feature space.

To visualize the clustering structure, pairwise projections of the ESI–MSI–LGP feature space are presented in Figure 9. The 77 core samples were divided into three clusters, including 24 samples in Cluster 1, 26 samples in Cluster 2, and 27 samples in Cluster 3. The three clusters show distinguishable but partially overlapping distributions in the projected feature space, indicating that the proposed strength-development indices capture different aspects of hardening behavior.

The pairwise projections indicate that ESI and MSI are positively associated, whereas MSI and LGP exhibit a clear negative relationship. This suggests that mixtures achieving a higher proportion of strength by 7 days generally have lower remaining LGP. Cluster 1 is mainly characterized by high ESI and MSI values, corresponding to rapid early- and mid-age strength development. Cluster 2 shows relatively low MSI and high LGP values, indicating that a larger proportion of strength gain occurs after 7 days. Cluster 3 is characterized by relatively low ESI but high MSI and low LGP, suggesting that strength development is limited within the first 3 days but accelerates markedly between 3 and 7 days. Although partial overlap exists among the clusters, their centroids occupy distinct regions in the ESI–MSI–LGP space.

**Figure 10:**
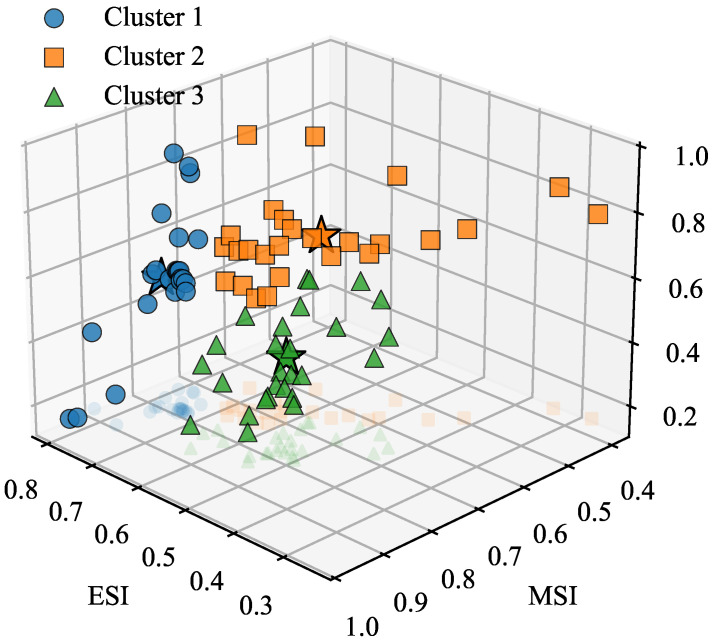
Three-dimensional visualization of strength-development pathways in the ESI–MSI–LGP feature space.

To further investigate the global cluster structure, the clustering results were visualized in the three-dimensional ESI–MSI–LGP feature space, as shown in Figure 10. Compared with the two-dimensional projections, the three-dimensional representation provides a more comprehensive view of the spatial relationships among the identified clusters. The three-dimensional visualization confirms that the identified pathways occupy different regions of the feature space, while also showing a continuous transition among them. The cluster centroids, indicated by stars in the figure, reflect distinct combinations of early-age strength attainment, mid-age strength attainment, and late-stage growth potential.

The statistical characteristics of the three clusters are summarized in Table 2. Cluster 1 was identified as the Fast-hardening pathway because it exhibited the highest ESI and MSI values. Cluster 2 was interpreted as the Delayed-hardening pathway due to its highest LGP value, indicating stronger late-stage strength-growth potential. Cluster 3 was classified as the Balanced-hardening pathway, characterized by relatively low ESI but high MSI and low LGP, indicating accelerated strength development between 3 and 7 days.

### 4.3. Representative Strength Development Behaviors

**Figure 11:**
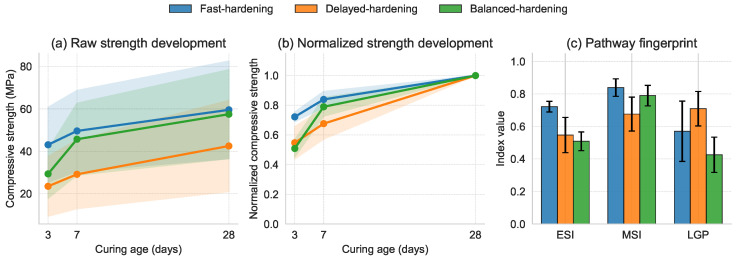
Representative strength-development characteristics of the identified pathways: (**a**) average compressive strength evolution at 3, 7, and 28 days, (**b**) normalized strength-development trajectories, and (**c**) pathway fingerprints based on ESI, MSI, and LGP.

Figure 11a visualizes the average strength-development trajectories at 3, 7, and 28 days. The Fast-hardening pathway exhibited the highest strength level throughout the curing period, indicating rapid early-age strength formation. The Delayed-hardening pathway showed the lowest strength level at early and middle ages, but continued to gain strength toward 28 days. The Balanced-hardening pathway showed a relatively low 3-day strength but developed rapidly between 3 and 7 days, approaching the Fast-hardening pathway at a later age.

To eliminate the influence of absolute strength level, the strength values were normalized by the corresponding 28-day compressive strength, as shown in Figure 11b. The Fast-hardening pathway achieved approximately 72% of its 28-day strength within the first 3 days and about 84% by 7 days, confirming its rapid early- and mid-age hardening behavior. The Delayed-hardening pathway reached approximately 55% and 68% of its 28-day strength at 3 and 7 days, respectively, indicating a slower strength-development process and a larger proportion of later-age strength gain. The Balanced-hardening pathway achieved approximately 51% of its 28-day strength at 3 days but increased to about 79% by 7 days, suggesting accelerated strength development during the 3–7-day period.

The pathway fingerprints in Figure 11c provide a compact representation of the three pathways in the ESI–MSI–LGP feature space. The Fast-hardening pathway is characterized by the highest ESI and MSI values, whereas the Delayed-hardening pathway exhibits the highest LGP value, reflecting stronger late-stage growth potential. In contrast, the Balanced-hardening pathway has relatively low ESI, high MSI, and the lowest LGP value, indicating that most of its post-3-day strength gain occurs before 7 days.

Overall, the differences among the pathways are primarily associated with the temporal pattern of strength development rather than the final strength level alone. Although the Fast-hardening pathway achieved the highest average 28-day strength, the normalized trajectories and pathway fingerprints show that the three pathways differ mainly in the timing of strength gain. Therefore, the identified pathways represent distinct macroscopic hardening patterns of one-part geopolymers rather than simply different strength grades.

**Figure 12:**
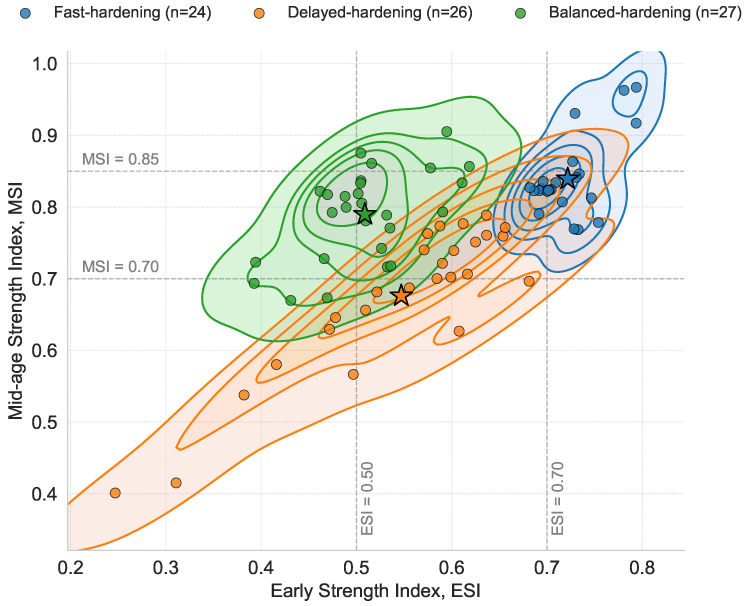
Pathway density landscape in the ESI–MSI feature space.

Figure 12 further reveals the spatial organization of the identified pathways in the ESI–MSI feature space, with the stars marking the centers of the respective pathways. Distinct density regions can be observed for the three pathways, indicating that the identified clusters correspond to different strength-development patterns. The Fast-hardening pathway is concentrated in the upper-right region, characterized by high ESI and MSI values. This confirms that mixtures in this pathway achieve a large proportion of their 28-day strength within the first week.

The Delayed-hardening pathway exhibits a more elongated distribution extending from the lower-left to the central region of the feature space, reflecting relatively slower early- and mid-age strength development. In contrast, the Balanced-hardening pathway is mainly located in the upper-central region, with moderate ESI but relatively high MSI values. This pattern suggests that these mixtures develop strength more slowly within the first 3 days but experience accelerated strength gain between 3 and 7 days.

Partial overlap can be observed among adjacent pathways, especially near the transitional regions between the Delayed-hardening and Balanced-hardening samples. This indicates that strength-development behavior evolves continuously rather than through strictly discrete categories. Nevertheless, the pathway centroids remain separated in the ESI–MSI space, supporting the use of three representative pathways to describe the main hardening patterns of one-part geopolymers.

### 4.4. Mix-Design Characteristics of Identified Pathways

**Figure 13:**
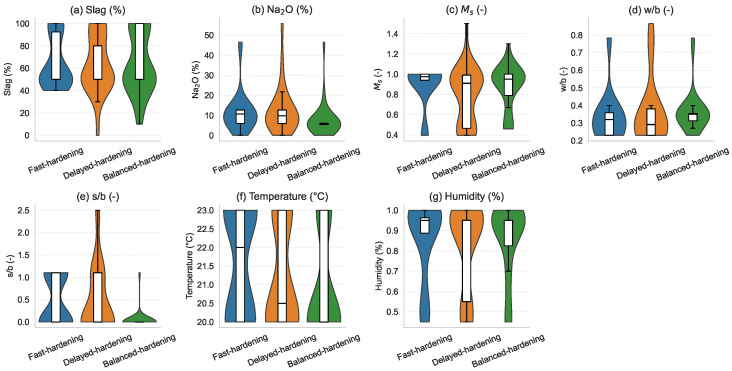
Distributions of mix-design variables among the identified strength-development pathways: (**a**) slag content, (**b**) Na_2_O dosage, (**c**) activator modulus ($M_{s}$), (**d**) water-to-binder ratio (w/b), (**e**) sand-to-binder ratio (s/b), (**f**) curing temperature, and (**g**) curing humidity.

To investigate the potential factors associated with pathway formation, the distributions of the seven mix-design variables among the identified pathways were compared, as shown in Figure 13. Overall, the distributions of most variables overlap among the three pathways, indicating that pathway formation cannot be attributed to a single mix-design parameter.

Among the investigated variables, the sand-to-binder ratio (s/b) shows the most noticeable difference among the three pathways. The Delayed-hardening pathway exhibits a wider s/b distribution and includes mixtures with relatively high s/b values, whereas the Balanced-hardening pathway is mainly concentrated at lower s/b values. This suggests that s/b may influence the apparent strength-development behavior by changing the binder volume fraction, packing structure, and interfacial characteristics.

The activator-related variables, including Na_2_O dosage and activator modulus ($M_{s}$), also show certain differences among the pathways, although substantial overlap remains. From a materials perspective, these variables are closely related to alkaline activation, precursor dissolution, and gel formation, and therefore may affect both early-age hardening and later-age strength growth. The water-to-binder ratio (w/b) also exhibits overlapping but distinguishable distributions, reflecting the role of water availability in dissolution, reaction kinetics, and pore-structure development.

In contrast, slag content, curing temperature, and humidity do not show clear separation among the three pathways in the present dataset. This may be partly related to the limited distribution range of some variables, particularly curing temperature, as most samples were cured under near-ambient conditions. Overall, the results suggest that the identified strength-development pathways are governed by the coupled effects of precursor composition, activator chemistry, water availability, aggregate-to-binder proportion, and curing conditions, rather than by any single dominant factor. The Kruskal–Wallis test indicated significant differences among the three pathways for Na_2_O dosage (H=10.595, p=0.0050) and s/b (H=10.742, p=0.0047), whereas the other mixture-design variables showed no statistically significant differences (p>0.05). One-way ANOVA additionally confirmed a significant difference in s/b (F=5.315, p=0.0070). These results support the observed differences while confirming substantial overlap among the pathways for most variables.

**Figure 14:**
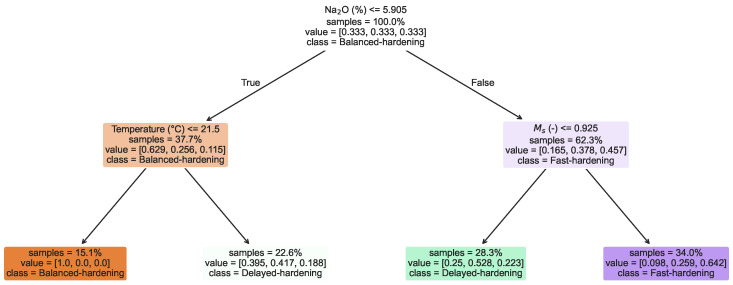
Interpretable pathway-identification rules extracted using a pruned decision tree model.

Using the decision-tree model described in Section 3, simplified pathway-identification rules were extracted from the seven mix-design variables. The resulting decision tree is presented in Figure 14. The first split was governed by Na_2_O dosage, suggesting that the alkali content plays an important role in differentiating strength-development pathways. Subsequent splits involved the curing temperature and activator modulus ($M_{s}$), indicating that pathway formation may be associated with the combined effects of activator chemistry and curing conditions. The pruned decision tree contained four terminal leaves and achieved a held-out accuracy of 0.542 and a weighted F1-score of 0.538. Given the moderate classification performance, the unsupervised origin of the pathway labels, and the limited size of the core dataset, the extracted rules should be interpreted as exploratory associations rather than predictive design rules. These results indicate that the identified pathways cannot be fully explained by a few threshold-based rules, but are likely governed by the coupled effects of multiple mix-design variables, including alkali dosage, activator modulus, water availability, aggregate-to-binder proportion, and curing conditions.

### 4.5. Strength-Development Map for One-Part Geopolymers

**Figure 15:**
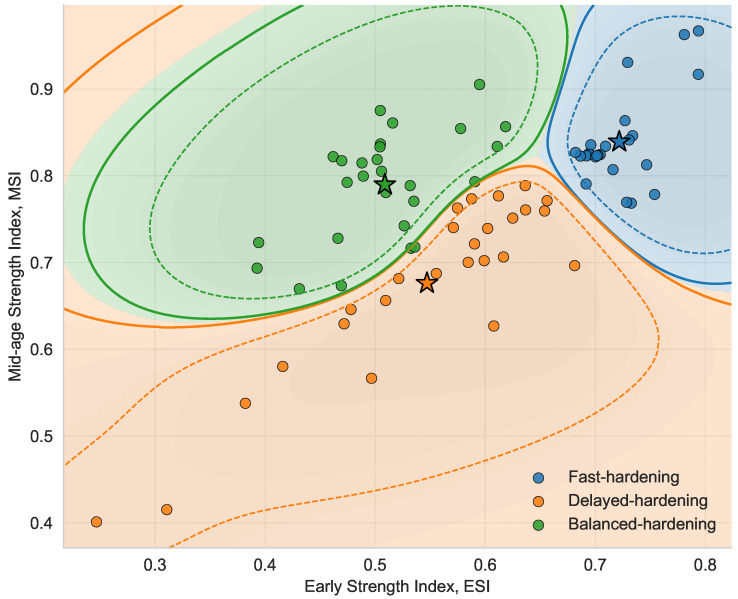
Smooth probability-based strength-development map of one-part geopolymers in the ESI–MSI feature space.

Based on the identified pathways, a smooth probability-based strength-development map was constructed in the ESI–MSI feature space, as shown in Figure 15. The map provides a two-dimensional visualization of the characteristic regions associated with the Fast-hardening, Delayed-hardening, and Balanced-hardening pathways, with the stars indicating the centroid of each pathway.

The Fast-hardening pathway occupies the upper-right region of the map and is characterized by high ESI and MSI values. Mixtures located in this region can achieve a large proportion of their 28-day strength within the first week of curing and are therefore suitable for applications requiring rapid early-age strength development. In contrast, the Delayed-hardening pathway mainly occupies the lower and central-left regions of the map, where both ESI and MSI are relatively low. These mixtures exhibit slower early- and mid-age strength development, but tend to retain greater late-stage growth potential. The Balanced-hardening pathway is mainly distributed in the upper-central-to-left region of the map, characterized by moderate ESI but relatively high MSI values. This indicates that such mixtures do not develop strength very rapidly within the first 3 days, but undergo accelerated strength gain between 3 and 7 days.

An important observation from Figure 15 is that pathway separation becomes much clearer in the strength-development feature space than in the original mix-design space. This finding further supports the conclusion that pathway formation is governed by the combined effects of multiple design variables rather than any single parameter. From a practical perspective, the proposed map provides a useful visualization tool for one-part geopolymer systems. By calculating the ESI and MSI values of a new mixture, its likely strength-development pathway can be approximately identified, thereby assisting mixture proportioning, curing-strategy selection, and performance optimization. Because the original clustering was performed in the three-dimensional ESI–MSI–LGP space, the ESI–MSI map should be interpreted as an approximate two-dimensional visualization tool. It is useful for preliminary pathway identification after early- and mid-age strength data are made available, but it does not fully replace the original GMM classification. Therefore, Figure 15 should be interpreted as an approximate two-dimensional projection for preliminary pathway identification after the required strength data become available, rather than as a complete representation or replacement of the original GMM classification.

## 5. Discussion

### 5.1. Origin of Strength-Development Pathways

The identification of the Fast-hardening, Delayed-hardening, and Balanced-hardening pathways demonstrates that strength evolution in one-part geopolymers cannot be fully represented by a single compressive strength value. Instead, mixtures with different temporal strength-development patterns can be distinguished using the proposed ESI–MSI–LGP feature space. The Fast-hardening pathway exhibited the highest early- and mid-age strength attainment, with average ESI and MSI values of 0.722 and 0.839, respectively. This indicates that mixtures in this pathway achieved a large proportion of their 28-day strength within the first week.

In contrast, the Delayed-hardening pathway showed lower ESI and MSI values, but the highest LGP value. This suggests that its strength development was relatively slow during the early and mid-age stages, while a larger fraction of strength gain occurred after 7 days. The Balanced-hardening pathway was characterized by relatively low ESI, high MSI, and the lowest LGP values, indicating that strength development was limited within the first 3 days but accelerated markedly between 3 and 7 days.

These observations indicate that the identified pathways mainly reflect differences in the timing of strength gain rather than simply differences in final strength level. The clusters should therefore be interpreted as representative regions within the strength-development space rather than strictly discrete categories. The partial overlap among pathways in the ESI–MSI feature space further suggests that strength-development behavior evolves continuously across mixtures.

### 5.2. Potential Factors Associated with Pathway Formation

The comparison of mix-design variables indicates that pathway formation is associated with the coupled effects of multiple parameters rather than a single dominant factor. Among the investigated variables, the sand-to-binder ratio (s/b) showed the most noticeable difference among the three pathways. This suggests that s/b may influence the apparent strength-development behavior by changing the binder volume fraction, packing structure, paste thickness, and interfacial characteristics. However, its effect should be interpreted mainly as a physical dilution and packing effect rather than a direct chemical control on geopolymerization.

Activator-related variables, including the Na_2_O dosage and activator modulus ($M_{s}$), also showed certain differences among pathways. From a materials perspective, these variables are closely related to alkaline activation, precursor dissolution, and gel formation, and therefore may affect both early-age hardening and later-age strength growth. The water-to-binder ratio (w/b) may further influence strength development by regulating water availability, reaction kinetics, and pore-structure evolution. Nevertheless, substantial overlap was observed for most of the mix-design variables, indicating that similar mixture proportions may still follow different strength-development trajectories.

The decision tree analysis provides simplified pathway-identification rules based on the mix-design variables. However, the extracted rules should be interpreted as exploratory rather than deterministic, because the strength-development pathways were derived from coupled temporal strength indices rather than directly from mix-design parameters. Overall, these results suggest that pathway formation is a multivariate process governed by the combined effects of precursor composition, activator chemistry, water availability, aggregate-to-binder proportion, and curing conditions.

### 5.3. Engineering Implications and Limitations

Relying solely on 28-day compressive strength may overlook important differences in early- and mid-age performance. For example, mixtures with comparable final strength may differ substantially in the proportion of strength gained within the first 3 or 7 days. The proposed pathway-oriented framework provides a practical approach for distinguishing such differences. Fast-hardening mixtures may be more suitable for rapid construction, prefabrication, or early demolding applications, whereas Delayed-hardening mixtures may be acceptable in cases where long-term strength development is more important than early-age strength. Balanced-hardening mixtures represent systems with delayed initial hardening but rapid strength gain between 3 and 7 days.

The ESI–MSI strength-development map provides a useful visualization tool for approximate pathway identification. By calculating the ESI and MSI values of a new mixture, its likely strength-development pathway can be preliminarily inferred. However, because the original clustering was performed in the three-dimensional ESI–MSI–LGP space, the two-dimensional map should be interpreted as a projection-based visualization rather than a complete representation of the full clustering boundary.

Several limitations should be acknowledged. First, the database was compiled from different literature sources, and variations in raw materials, curing regimes, specimen preparation, and testing protocols may introduce heterogeneity. Unequal contributions from individual publications and differences in the availability of multi-age measurements may introduce source and selection biases. These factors limit population-level inference from the present pathways. Second, the pathway identification was based mainly on 3-day, 7-day, and 28-day compressive strength data, while intermediate-age measurements such as 14-day strength were limited. Third, the proposed indices and pathways were derived from macroscopic strength data, and the underlying reaction mechanisms were inferred rather than directly observed. Moreover, the clustering was based on 77 complete literature-derived records. The identified pathways should therefore be regarded as representative patterns within the compiled dataset, and their generality should be evaluated using larger independent datasets.

Future work should expand the database with more complete and denser multi-age strength records and integrate calorimetry, reaction-kinetic modeling, and microstructural characterization, such as XRD, SEM, and FTIR. These efforts would help determine whether the empirically identified pathways correspond to distinct reaction-rate regimes and phase-evolution processes and support the development of predictive models linking mixture design, reaction mechanisms, and strength-development pathways.

## 6. Conclusions

This study presents a pathway-oriented framework for characterizing strength-development behavior in one-part geopolymers (OPGs). A compiled database of OPG mixtures was established from the published literature, and three dimensionless indices, namely the Early Strength Index (ESI), Mid-age Strength Index (MSI), and Late-stage Growth Potential (LGP), were introduced to quantify temporal strength evolution. Based on the 77 core samples containing complete 3-day, 7-day, and 28-day compressive strength records, Gaussian Mixture Model (GMM) clustering identified three representative strength-development pathways: Fast-hardening, Delayed-hardening, and Balanced-hardening. The key findings are as follows:

- The Fast-hardening pathway exhibited the highest early- and mid-age strength attainment, with average ESI and MSI values of 0.722 and 0.839, respectively. This indicates that mixtures in this pathway achieved a large proportion of their 28-day strength within the first week.
- The Delayed-hardening pathway showed lower ESI and MSI values but the highest LGP value of 0.709, indicating that a larger fraction of strength gain occurred after 7 days. In contrast, the Balanced-hardening pathway was characterized by relatively low ESI, high MSI, and the lowest LGP values, suggesting accelerated strength development between 3 and 7 days.
- The comparison of mix-design variables indicated that pathway formation is associated with the coupled effects of multiple parameters rather than a single dominant factor. The sand-to-binder ratio (s/b) showed the most noticeable statistical difference among pathways, while activator chemistry, water availability, precursor composition, and curing conditions may jointly influence the observed strength-development behavior.
- The proposed ESI–MSI-based strength-development map provides a practical visualization tool for approximate pathway identification and mixture selection. Since the original clustering was performed in the three-dimensional ESI–MSI–LGP feature space, the two-dimensional map should be interpreted as a projection-based tool rather than a complete representation of the full clustering boundary.

Overall, this framework shifts the focus from conventional strength prediction at a single curing age to the characterization of temporal hardening behavior. The results demonstrate that OPG mixtures with similar final strength levels may follow different strength-development pathways, highlighting the importance of considering early-, mid-, and later-age strength evolution in mixture design. Future work should expand the database with more complete multi-age strength records, incorporate microstructural and reaction-kinetic characterization, and develop predictive models linking mixture design, reaction mechanisms, and strength-development pathways.

## Figures and Tables

**Table 1:** Summary statistics of the compiled database for one-part geopolymers.

Variable	Unit	Samples	Min	Max	Mean	Std
CS_3d_	MPa	107	1.0	77.196	30.833	16.369
CS_7d_	MPa	183	0.2	83.323	37.353	20.350
CS_14d_	MPa	38	1.799	58.572	33.037	17.239
CS_28d_	MPa	183	0.3	105	48.903	25.433

**Table 2:** Statistical characteristics of the three GMM-identified strength-development pathways. Values are reported as mean ± standard deviation.

Cluster	Pathway	Samples	ESI	MSI	LGP
1	Fast-hardening	24	0.722 ± 0.033	0.839 ± 0.054	0.570 ± 0.185
2	Delayed-hardening	26	0.547 ± 0.108	0.676 ± 0.104	0.709 ± 0.106
3	Balanced-hardening	27	0.509 ± 0.058	0.790 ± 0.063	0.426 ± 0.108

## Data Availability

The data presented in this study are available upon reasonable request from the corresponding author, as the data are currently being used in ongoing follow-up research.

## Abbreviations

The following abbreviations are used in this manuscript:

OPG	One-Part Geopolymer
GMM	Gaussian Mixture Model
AIC	Akaike Information Criterion
BIC	Bayesian Information Criterion
ANOVA	Analysis of Variance
ESI	Early Strength Index
MSI	Mid-Age Strength Index
LGP	Late-Stage Growth Potential
CS	Compressive Strength
w/b	Water-to-Binder Ratio
s/b	Sand-to-Binder Ratio
$M_{s}$	Activator Modulus
RH	Relative Humidity
